# Positive airway pressure settings do not predict outcomes of maxillomandibular advancement surgery in the treatment of obstructive sleep apnea

**DOI:** 10.1007/s10006-025-01421-8

**Published:** 2025-06-21

**Authors:** John M. Nathan, Douglas F. Werkman, Cameron C. Lee, Zachary S. Peacock, Anita V. Shelgikar, Jeffrey J. Stanley, Hossein E. Jazayeri, Jonathan P. Troost, Sharon Aronovich

**Affiliations:** 1 Department of Oral and Maxillofacial Surgery and Hospital Dentistry, University of Michigan, Ann Arbor, MI United States of America; 2 University of Michigan Medical School, Ann Arbor, MI United States of America; 3 Department of Oral and Maxillofacial Surgery, University of Maryland, Baltimore, MD United States of America; 4 Department of Oral and Maxillofacial Surgery, Massachusetts General Hospital and Harvard School of Dental Medicine, Boston, MA United States of America; 5 Department of Neurology, University of Michigan, Ann Arbor, MI United States of America; 6 Department of Otolaryngology - Head and Neck Surgery and Neurology, University of Michigan, Ann Arbor, MI United States of America; 7 Michigan Institute for Clinical & Health Research, University of Michigan, Ann Arbor, MI United States of America

**Keywords:** Maxillomandibular advancement, Obstructive sleep apnea, Therapeutic CPAP settings, Pharyngeal critical closing pressure, PCrit, MMA

## Abstract

**Purpose:**

The purpose of this study was to determine if the therapeutic continuous positive airway pressure (CPAP) setting identified during pre-surgical CPAP titration polysomnogram (PSG) is predictive of surgical success after MMA in adults with OSA.

**Methods:**

This multi-institutional retrospective study evaluated adults treated for OSA with MMA between 2000 and 2020 at Michigan Medicine and Massachusetts General Hospital. Patients included were those with a diagnosis of moderate-severe OSA (AHI > 15), a pre-operative CPAP titration PSG, and available pre-operative and postoperative PSGs. The primary predictor variable was the recommended CPAP setting from the pre-op titration PSG. Secondary predictor variables included preoperative oxygen saturation nadir, and hypoxic burden. The primary outcome variable was surgical success (achieving an AHI of < 20 and >/=50% reduction in AHI). The secondary outcome variable was change in AHI. Logistic and linear regression were used to model outcomes. Unadjusted and bivariable adjusted models were performed for predictors. Significance was set to *P* <.05.

**Results:**

31 subjects were included in this study (25 males, average age 42.9 ± 9.67 years). Mean pre-surgical CPAP titration setting was 10.4 cm H2O. On mixed model regression, recommended CPAP setting did not predict surgical success (adjusted ß = 0.93, *P* =.56), nor change in AHI (adjusted ß = 0.35, *P* =.51). In the latter model, age was found to be statistically significant, with each increased year of age increasing the postop AHI by 0.51/hr (*P* =.004). Hypoxic burden was also significant and positively correlated with AHI (adjusted ß = 0.90, *P* =.02).

**Conclusion:**

MMA remains an effective treatment modality for carefully selected patients with OSA, however, pre-operative therapeutic CPAP values were not a reliable predictor of surgical success.

**Supplementary Information:**

The online version contains supplementary material available at 10.1007/s10006-025-01421-8.

## Introduction

Obstructive sleep apnea (OSA) is a disorder characterized by repetitive upper airway obstruction during sleep that is associated with multiple comorbidities including excessive daytime somnolence, impaired cognition, and increased risk for cardiovascular disease [1]. OSA prevalence has been reported to range from 9 to 38% of the overall population with an even higher prevalence in older populations [2]. Continuous positive airway pressure (CPAP) is the first line treatment for OSA and has shown to decrease cardiovascular related risks and decrease daytime somnolence [3–5]. However, CPAP tolerance and compliance varies widely depending on many factors including severity of OSA, type of equipment, nasal obstruction, xerostomia, and claustrophobia [6–9]. In addition, mandibular advancement devices (MAD) should be considered for mild to moderate OSA prior to surgical intervention. Patients that are CPAP and MAD intolerant should undergo a multidisciplinary evaluation to determine the most effective treatment options individualized to that patient.

Surgical options for the management of OSA include nasal surgery, soft tissue surgery, nerve stimulation, skeletal surgery, or a combination of approaches. Maxillomandibular advancement (MMA) is a skeletal option commonly offered by oral and maxillofacial surgeons (OMFS) that surgically advances the maxilla and mandible. The primary goal of the procedure is to increase upper airway volume and decrease airway collapsibility. By increasing the upper airway volume after MMA, airway flow will increase per Poiseuille’s law.

High success rates of MMA, 55 − 90%, are attributed to expansion of the upper airway at multiple levels [10–12]. The treatment of OSA with MMA has overall favorable results, however, there remains patients that have significant residual disease or minimal response after surgery. In addition, MMA is associated with increased complication rates and increased morbidity compared to traditional orthognathic surgery [13, 14]. Given the significant morbidity and irreversible nature of MMA, patient selection and predictors of success are critical. Factors associated with decreased success include increasing patient age, neck circumference, central apnea index, (CAI), and superior posterior airway space. Lower body mass index (BMI), female gender, lower starting apnea hypopnea index (AHI), and greater advancement distance are associated with increased success rates [10, 15, 16]. Using a combination of these variables in a predictive model to aid with patient selection has not been reported in the literature.

The use of pharyngeal critical closing pressure (PCrit), the amount of pressure needed to keep the pharyngeal airway from collapsing, has shown to correlate with therapeutic CPAP settings [17, 18]. In addition, PCrit has demonstrated modest correlation with increasing severity of OSA [19]. Patients with lower therapeutic CPAP settings thus require less pressure to stent the airway open thus MMA may have a higher probability of success. To date there have not been studies reporting post-MMA PCrit values, however, mandibular advancement devices have demonstrated the ability to decrease the PCrit values [20].

The purpose of this study was to evaluate multi-institutional data to determine if CPAP setting from a pre-surgical CPAP titration polysomnogram (PSG) is predictive of success of MMA (i.e. AHI reduction of 50% or more and AHI < 20) in adults with OSA. We hypothesized that lower CPAP settings would predict a more effective response to MMA. The aims of this study include:

1. Determine if a correlation exists between pre-operative CPAP setting and surgical success or resolution of OSA (i.e. AHI < 5) post-operatively.
2. Compare success rates between patients undergoing MMA with high and low pre-operative CPAP settings (i.e., ≥ 8 vs. <8 cm H_2_O).
3. Examine the effect of therapeutic CPAP settings on surgical success in a multivariate analysis of surgical predictors.

## Methods

### Study design and sample

This was a multi-institutional retrospective cohort study of patients undergoing MMA for OSA at Michigan Medicine (MM) or Massachusetts General Hospital (MGH) between the years 2000 and 2020. Adult patients were included as participants if they had a diagnosis of moderate or severe OSA (AHI > 15), had a pre-operative CPAP titrating PSG within 5 years of surgery, underwent MMA, and had preoperative and postoperative PSG. Patients with a craniofacial syndrome, predominant central sleep apnea (Central Apnea Index or CAI ≥ 5), and those that had additional airway operations after MMA prior to a postoperative PSG were excluded. Total MMA cases performed over the specified period at MGH included 110 patients and MM included 30 patients. After review, 22 patients from MGH and 9 patients from MM met the inclusion criteria for this study. This study was approved by both the University of Michigan and Massachusetts General Hospital (Protocol #2021P001087) institutional review boards.

### Surgical procedures

All MMAs were performed by attending surgeons with the assistance of resident surgeons. MMA was planned with either traditional model surgery or computer-assisted surgical simulation. The operation included Le Fort I maxillary osteotomy and bilateral sagittal split ramus osteotomies of the mandible. Participants with maxillary advancement ≥ 6 mm generally underwent bone grafting of the anterior maxilla between the buttresses with tricortical iliac crest allograft secured with positional screws. Mandibular osteotomies underwent fixation with either bicortical screws, titanium alloy plates and monocortical screws, or a combination of both. In cases with counterclockwise rotation of the occlusal plane, the latter was supplemented with bicortical screws to achieve rigid fixation, or an interpositional allograft block rigidly fixed between the proximal and distal segments. Participants undergoing adjunctive procedures, genioglossus advancement (GGA), and uvulectomy were included. GGA was performed via the box technique or via a modified sliding genioplasty including the tubercles if a chin advancement was deemed esthetically beneficial to achieve a straight profile. No patient required tracheostomy. No patient required ICU admission.

### Study variables

Polysomnographic, demographic, and surgical variables were evaluated. The primary predictor variable was the recommended therapeutic CPAP setting (cm of H_2_O) from the preoperative titration PSG. Secondary predictor variables were preoperative oxygen saturation nadir (min SpO2) and preoperative AHI. Exploratory predictor variables included age, body mass index (BMI), change in BMI, and percent time spent < 88% oxygen saturation. For the purposes of this study, percent time spent < 88% oxygen saturation is used as a surrogate for hypoxic burden and will be referred to as hypoxic burden.

The primary outcome variable was surgical success, defined as achieving an AHI of < 20 events/hour and a 50% or greater reduction in AHI on follow-up PSG; the follow-up PSGs were done 5.7 +/- 2.8 months after surgery. The secondary outcome variable was change in AHI. The exploratory outcome variable was resolution of OSA (AHI < 5) post-operatively.

### Statistical analysis

Descriptive statistics were provided for the combined cohort using means, standard deviations, medians, interquartile ranges, and ranges for continuous variables, and using frequencies and percentages for categorical variables. Comparisons by site were made using Kruskall-Wallis tests for continuous and chi-square tests for categorical variables. Logistic regression was used to model dichotomous outcomes (i.e., surgical success) and linear regression for continuous outcomes (i.e., mean change in AHI). Unadjusted models were performed for the following predictors: CPAP value, CPAP high vs. low (i.e., ≥ 8 vs. <8), preop O2 nadir, percentage time spent with an oxygen saturation < 88%, age, BMI, and change in BMI. Bivariable adjusted models were repeated for each covariate above adjusted for preop AHI. Analyses were performed in SAS V9.4 (SAS institute Inc., Cary, NC, USA).

## Results

### Demographics

A total of 31 participants were included in this study including 25 males with a mean age of 42.9 ± 9.67 (range 26 to 56) years. Nine participants were from MM and 22 from MGH. Mean time from pre-operative titration PSG to date of surgery was 33.8 days, and mean time from surgery to post-operative PSG was 5.7 months ± 2.8. All 31 participants had pre-op attended PSG performed. 27 participants had post-op attended PSG and 4 participants had a home sleep apnea test (HSAT). See Table 1.

All 22 participants treated at MGH received MMA and genioglossus advancement (GGA). Of the 9 MM participants, 1 received MMA alone, 2 received MMA + GGA, 3 received MMA + uvulectomy, and 3 received MMA + GGA + Uvulectomy.

The intended maxillomandibular advancement ranged from 8 to 12 mm. The average BMI was 30.5 kg/m^2^ (5.16, 26.6–41.8), the average change in BMI was − 0.3 kg/m^2^ (3.11, -5.2-10.90). No patient had significant hardware failure or mal-union that changed skeletal positioning at the time of post-operative PSG.

### Predictor (independent) variables

Participants had a mean pre-surgical CPAP titration PSG recommended setting of 10.4 cm of H2O (SD 3.34, Range 5–17). The mean preoperative min SpO2 was 80.0% (SD 9.56, Range 57–94). The average preoperative AHI was 48.1/hr (SD 23.02, Range 15.3–98.0).

### Outcome (dependent) variables

Surgical success was attained in 24 of the 31 participants (77%) and the mean decrease in AHI was 36.7/hr (SD 22.23), *P =.25.* Resolution of OSA was achieved in 9 participants (29%). See Table 1.

Surgical success was attained in 5 of 7 (71.4%) of participants with recommended PAP settings < 8 cm H2O. Surgical success was attained in 16 of 24 (66.7%) of participants with PAP settings > 8 cm H2O (chi-square = 0.056, *P* =.81).

### CPAP value and surgical success

On mixed model regression analysis, therapeutic CPAP setting did not predict surgical success (adjusted β = 0.93, *P* =.56). No significant effect was seen between the following predictor variables and surgical success: min SpO2, hypoxic burden, BMI, change in BMI, or preoperative AHI. Age had statistically significant inverse correlation with surgical success. See Table 2.

On further mixed model regression, CPAP setting did not predict change in AHI (adjusted β = 0.35 *P* =.51). The following predictor variables did not have a significant effect on change in AHI: min SpO2, BMI, change in BMI, or preoperative AHI. This model showed age to have a significant effect; each increased year of age increased the post-operative AHI by 0.44/hr, *P* =.02). Additionally, hypoxic burden was found to be significant and positively correlated in the adjusted model (adjusted β = 0.90, *P* =.02). CPAP setting also did not predict the likelihood of achieving an AHI < 5 post-operatively (adjusted β = 1.03, *P* =.83). See Table 3.

No significant effect was seen between the following predictor variables and resolution of OSA: minSpO2, hypoxic burden, age, BMI, change in BMI, or preoperative AHI. Each increased year of age reduced the odds of achieving OSA resolution by 0.87 (*P =.01*). See Table 4.

Bonferroni corrected p-value threshold was 0.017 (0.05/3) and p-values between 0.017 and 0.05 should be interpreted with caution.

Multivariable analysis did not demonstrate significant association of therapeutic CPAP values with adjusting for the variable combinations reported. See Table 5.

Sub-group analysis of patients with MMA alone, MMA with GGA, MMA with GGA and uvulectomy, and MMA with uvulectomy was performed. Pre-op AHI mean for MMA alone 45.98, MMA with GGA 54.45, MMA with GGA and uvulectomy 35.70, MMA with uvulectomy 73 with *p* =.27. Surgical success, defined by AHI < 15 and 50% reduction, for MMA alone 65.22% (15/23), MMA with GGA 50% (1/2), MMA with GGA and uvulectomy 100% (3/3), MMA with uvulectomy 67.74% (2/3) with *p* =.62. Mean change in AHI for MMA alone − 34.55, MMA with GGA − 41.25, MMA with GGA and uvulectomy − 28.27, and MMA with uvulectomy − 58.43 with *p* =.38.

Mean CPAP value for patients with and without surgical success defined as AHI < 20 and 50% reduction in AHI was 10.2 and 11.0 respectively. See Supplemental Table 1.

## Discussion

The purpose of this pilot study was to evaluate the association between pre-operative therapeutic CPAP settings and surgical success of MMA, defined as an AHI of < 20 and a reduction in AHI of 50% or greater on post-operative PSG. We hypothesized that lower therapeutic CPAP levels would predict a more effective response to MMA surgery. Our results did not support the use of therapeutic CPAP settings as a predictor of surgical success, reduction in AHI, or OSA resolution for patients undergoing MMA for the treatment of moderate to severe OSA. In addition, CPAP settings did not demonstrate a statistically significant association with the outcomes of interest on multivariate analysis. However, our study provides additional evidence that MMA has a high overall surgical success rate (77%), increased patient age is associated with decreased improvements in AHI, and lower pre-operative AHI is associated with increased surgical success. In addition, hypoxic burden on bivariable adjustment was found to be positively associated with changes in post-operative AHI.

The pathophysiology of obstructive sleep apnea is a dynamic process with multiple contributing factors. Because of this complex relationship, identification of direct associations between potential predictors and outcomes is challenging. Previous studies have demonstrated the association between PCrit and therapeutic CPAP values [17–19]. In addition, studies have also shown the loose association between PCrit and OSA severity [19]. However, limited studies exist directly measuring post-MMA PCrit and therapeutic CPAP values. This has likely not been studied in depth due to the high overall success of the procedure, however, post-MMA computational fluid dynamics studies have demonstrated improvements in airflow dynamics that suggest improvement in PCrit values post-MMA [21–23]. Studies indirectly related to MMA and PCrit response have shown that mandibular advancement devices (MAD) can lower therapeutic CPAP pressures and PCrit [20, 24]. However, therapeutic CPAP pressures and PCrit have not consistently been associated with AHI [25]. In addition, there are a lack of studies demonstrating a direct correlation between PCrit and OSA related risks such as cardiovascular disease and cerebrovascular disease. In our study we use therapeutic CPAP pressure as a surrogate for PCrit to evaluate its utility as a predictor of MMA success. However, as reported, our study did not show that therapeutic CPAP pressure was a predictor of surgical outcomes of MMA.

Our study has limitations that may prevent determining if therapeutic CPAP settings can be used as a predictor for MMA success including limited sample size, potential confounding variables, and retrospective analysis. Our study is multi-institutional; however, the overall sample size is low with potentially confounding factors including variability of advancement distance and genioglossus advancement inclusion. MMA advancement distance has been shown to affect AHI while genioglossus advancement has had mixed results thus potentially masking the association between CPAP settings and post-intervention outcomes [26–28]. Studies have shown that MMA with GGA compared to MMA alone have not demonstrated statistically significant differences in post-operative AHI [29]. Mitigation of these areas include prospective studies with increased cohort size and standardized treatment protocols across all patients. Additional studies analyzing the association between PCrit, therapeutic CPAP settings, and AHI are needed with and without intervention.

Predicting an individual’s probability of success undergoing MMA remains an important topic of research due to the associated morbidity and irreversible nature of the procedure. In comparison to traditional orthognathic surgery, MMA is associated with increased rate of complications including dysesthesia, wound dehiscence, and infection [14]. Continuing to increase overall success rates whether its evolving surgical techniques or improving patient selection will help to minimize the number of patients that undergo MMA without significant improvement of their OSA. Further identification of predictors of MMA success could lead to the use of artificial intelligence and machine learning (AI/ML) to more precisely select surgical candidates. AI/ML has demonstrated promising results with screening patients for OSA with comparable results to traditional validated questionnaires [30]. However, utilization of AI/ML to predict MMA success will require large multi-institutional cohorts with standardized protocols. An alternative approach to predicting MMA success may be the use of drug induced sleep endoscopy (DISE). DISE directed soft tissue surgical interventions for treatment of patients with OSA has had mixed results with lack of quality data to reach a conclusion [31, 32]. When used for predicting MMA outcomes, preliminary study by Zhou et al. demonstrated that DISE may have a role in selecting patients undergoing MMA and identified posteriorly positioned epiglottis having a potentially negative correlation with MMA success [33]. Alternative areas that may provide insight include the use of pre-operative CBCT in combination with computational fluid dynamics. Studies have demonstrated improvements in airflow and decreased resistance in post-MMA patients [21, 34]. Prospective studies investigating these methods are needed to determine its role in patient selection.

Overall, our study demonstrated that maxillomandibular advancement is an effective treatment modality for carefully selected patients with moderate to severe OSA, however a variable spectrum of treatment responses may be seen on post-surgical polysomnograms. Therapeutic CPAP setting was not identified as a predictor of MMA success. Additional studies are needed to identify predictive variables to improve MMA success.

**Table 1:** Descriptive statistics by site

	Site			
Characteristic	Mass Gen	Michigan	Overall	*p*-value
CPAP Value				0.2168
Mean (SD)	10.4 (3.86)	10.3 (2.00)	10.4 (3.39)	
Median (IQR)	10.0 (7.00 to 14.00)	10.0 (9.00 to 12.00)	10.0 (8.00 to 13.00)	
Range	5.00 to 17.00	7.00 to 13.00	5.00 to 17.00	
N	22	9	31	
**CPAP: high vs. low**,** n (%)**				
Low (< 8 mm H20)	6 (27)	1 (11)	7 (23)	
High ( > = 8 mm H20)	16 (73)	8 (89)	24 (77)	
**Preop O2 nadir**				**0.91**
Mean (SD)	80.1 (8.95)	79.8 (11.44)	80.0 (9.56)	
Median (IQR)	83.0 (75.00 to 86.00)	84.0 (79.00 to 87.00)	83.5 (75.00 to 86.00)	
Range	59.00 to 94.00	57.00 to 91.00	57.00 to 94.00	
N	22	9	31	
**O2 time spent < 88% saturation**				**0.42**
Mean (SD)	2.8 (5.78)	0.7 (1.85)	2.1 (4.88)	
Median (IQR)	0.0 (0.00 to 3.55)	0.0 (0.00 to 0.10)	0.0 (0.00 to 1.00)	
Range	0.00 to 22.60	0.00 to 5.60	0.00 to 22.60	
N	22	9	31	
**Age (years)**				**0.05**
Mean (SD)	40.6 (10.19)	48.6 (5.32)	42.9 (9.67)	
Median (IQR)	41.0 (32.00 to 48.00)	49.0 (47.00 to 53.00)	44.0 (34.00 to 53.00)	
Range	26.00 to 56.00	38.00 to 55.00	26.00 to 56.00	
N	22	9	31	
**BMI**				**0.20**
Mean (SD)	30.0 (5.06)	31.9 (5.52)	30.5 (5.16)	
Median (IQR)	30.2 (26.00 to 33.00)	32.8 (29.30 to 34.95)	30.9 (26.60 to 34.40)	
Range	20.50 to 41.80	21.60 to 39.80	20.50 to 41.80	
N	22	9	31	
**Preop AHI**				**0.43**
Mean (SD)	46.1 (22.61)	53.1 (24.63)	48.1 (23.02)	
Median (IQR)	43.2 (26.80 to 66.00)	47.6 (38.50 to 61.30)	44.5 (28.10 to 66.00)	
Range	15.30 to 87.50	20.00 to 98.00	15.30 to 98.00	
N	22	9	31	
**Maxillary Advancement**				**0.01**
Mean (SD)	11.1 (2.48)	7.4 (2.89)	10.0 (3.07)	
Median (IQR)	12.0 (11.00 to 12.00)	7.0 (6.50 to 8.40)	12.0 (7.00 to 12.00)	
Range	4.00 to 14.00	2.90 to 13.10	2.90 to 14.00	
N	22	9	31	
**Mandibular Advancement**				**0.07**
Mean (SD)	11.6 (1.47)	13.5 (2.93)	12.2 (2.12)	
Median (IQR)	12.0 (12.00 to 12.00)	13.5 (11.30 to 15.20)	12.0 (11.30 to 12.80)	
Range	6.00 to 14.00	8.70 to 18.50	6.00 to 18.50	
N	22	9	31	
**Surgery**,** n (%)**				**< 0.0001**
MMA	22 (100)	1 (11)	23 (74)	
MMA GGA	0 (0)	2 (22)	2 (6)	
MMA GGA Uv	0 (0)	3 (33)	3 (10)	
MMA Uv	0 (0)	3 (33)	3 (10)	
**Apnea-hypopnea index (AHI) (from CPAP)**,** of < 20 and 50% reduction in AHI**,** n (%)**				**0.3286**
No	6 (27)	1 (11)	7 (23)	
Yes	16 (73)	8 (89)	24 (77)	
**Apnea-hypopnea index (AHI) (from CPAP)**,** of < 15 and 50% reduction in AHI**,** n (%)**				**0.4445**
No	8 (36)	2 (22)	10 (32)	
Yes	14 (64)	7 (78)	21 (68)	
**Change in AHI**				**0.25**
Mean (SD)	-34.5 (23.38)	-42.1 (19.30)	-36.7 (22.23)	
Median (IQR)	-32.1 (-51.40 to -14.00)	-40.9 (-42.70 to -30.60)	-35.0 (-51.40 to -15.00)	
Range	-83.80 to -0.50	-75.60 to -15.00	-83.80 to -0.50	
N	22	9	31	
**Apnea-hypopnea index (AHI) (from CPAP)**,** of < 5**,** n (%)**				**0.1597**
No	14 (64)	8 (89)	22 (71)	
Yes	8 (36)	1 (11)	9 (29)	

**Table 2:** Logistic regression results of Apnea-hypopnea index (AHI) (from CPAP), of < 20 and 50% reduction in AHI

	Model			
Variable	Unadjusted		Bivariable pre-op AHI adjusted	
	OR [95% CI]	*p*-value	OR [95% CI]	*p*-value
CPAP Value	0.93 [0.72, 1.20]	0.58	0.93 [0.72, 1.20]	0.56
CPAP: High vs. low	1.52 [0.22, 10.30]	0.66	1.61 [0.23, 11.44]	0.63
Preop O2 nadir	1.04 [0.96, 1.14]	0.34	1.09 [0.96, 1.24]	0.19
O2 time spent < 88% saturation	0.85 [0.68, 1.05]	0.13	0.84 [0.67, 1.06]	0.15
Age (per 1 year)	0.82 [0.68, 0.98]	0.02	0.77 [0.61, 0.99]	0.03
BMI	0.96 [0.81, 1.13]	0.62	0.95 [0.79, 1.13]	0.53
Maxillary Advancement	0.63 [0.37, 1.07]	0.08	0.63 [0.37, 1.07]	0.08
Mandibular Advancement	1.37 [0.85, 2.20]	0.19	1.38 [0.85, 2.26]	0.19
Preop AHI	1.00 [0.97, 1.04]	0.85	---	---

**Table 3:** Linear regression results of change in AHI

	Model			
Variable	Unadjusted		Bivariable pre-op AHI adjusted	
	OR [95% CI]	*p*-value	OR [95% CI]	*p*-value
CPAP Value	-0.24 [-2.73, 2.25]	0.84	0.35 [-0.75, 1.45]	0.51
CPAP: High vs. low	10.07 [-9.42, 29.57]	0.29	0.96 [-7.98, 9.91]	0.82
Preop O2 nadir	1.09 [0.37, 1.82]	0.004	-0.42 [-0.92, 0.09]	0.10
O2 time spent < 88% saturation	0.85 [-1.05, 2.75]	0.36	0.90 [0.12, 1.69]	0.02
Age (per 1 year)	0.12 [-0.75, 0.99]	0.78	0.51 [0.17, 0.85]	0.004
BMI	-1.30 [-2.91, 0.31]	0.11	0.09 [-0.71, 0.89]	0.81
Maxillary Advancement	1.38 [-1.32, 4.08]	0.30	0.70 [-0.50, 1.89]	0.24
Mandibular Advancement	-3.53 [-7.27, 0.22]	0.06	-1.11 [-2.88, 0.67]	0.21
Preop AHI	-0.87 [-1.03, -0.71]	< 0.001	---	---

**Table 4:** Logistic regression results of Apnea-hypopnea index (AHI) (from CPAP), of < 5

	Model			
Variable	Unadjusted		Bivariable pre-op AHI adjusted	
	OR [95% CI]	*p*-value	OR [95% CI]	*p*-value
CPAP Value	1.02 [0.81, 1.29]	0.85	1.03 [0.81, 1.30]	0.83
CPAP: High vs. low	1.03 [0.16, 6.62]	0.97	0.97 [0.15, 6.49]	0.97
Preop O2 nadir	1.07 [0.96, 1.19]	0.23	1.06 [0.93, 1.22]	0.36
O2 time spent < 88% saturation	0.06 [0.00, 12.96]	0.30	0.02 [0.00, 17.12]	0.26
Age (per 1 year)	0.87 [0.78, 0.97]	0.01	0.87 [0.78, 0.97]	0.01
BMI	0.94 [0.79, 1.11]	0.44	0.93 [0.78, 1.12]	0.43
Maxillary Advancement	1.06 [0.81, 1.39]	0.65	1.06 [0.81, 1.38]	0.67
Mandibular Advancement	1.00 [0.69, 1.45]	0.99	1.01 [0.69, 1.49]	0.93
Preop AHI	0.99 [0.96, 1.03]	0.75	---	---

**Table 5:** Multivariable model results

outcomevar		Estimate [95% CI]	*p*-value
Apnea-hypopnea index (AHI) (from CPAP), of < 20 and 50% reduction in AHI	Multivariable: pre-op AHI and O2 nadir adjusted	0.92 [0.70, 1.20]	0.52
	Multivariable: pre-op AHI and Spo88 adjusted	0.84 [0.62, 1.15]	0.28
	Multivariable: pre-op AHI and age adjusted	0.97 [0.73, 1.30]	0.85
	Multivariable: pre-op AHI and BMI adjusted	0.94 [0.73, 1.23]	0.66
	Multivariable: pre-op AHI and Maxillary Advancement adjusted	0.93 [0.72, 1.19]	0.56
	Multivariable: pre-op AHI and Mandibular Advancement adjusted	0.97 [0.74, 1.26]	0.79
Change in AHI	Multivariable: pre-op AHI and O2 nadir adjusted	0.49 [-0.57, 1.55]	0.34
	Multivariable: pre-op AHI and Spo88 adjusted	0.59 [-0.54, 1.72]	0.29
	Multivariable: pre-op AHI and age adjusted	0.24 [-0.73, 1.21]	0.61
	Multivariable: pre-op AHI and BMI adjusted	0.32 [-0.84, 1.49]	0.57
	Multivariable: pre-op AHI and Maxillary Advancement adjusted	0.42 [-0.68, 1.51]	0.44
	Multivariable: pre-op AHI and Mandibular Advancement adjusted	0.27 [-0.83, 1.37]	0.61
Apnea-hypopnea index (AHI) (from CPAP), of < 5	Multivariable: pre-op AHI and O2 nadir adjusted	0.99 [0.77, 1.27]	0.92
	Multivariable: pre-op AHI and Spo88 adjusted	0.99 [0.72, 1.36]	0.96
	Multivariable: pre-op AHI and age adjusted	1.09 [0.81, 1.46]	0.57
	Multivariable: pre-op AHI and BMI adjusted	1.07 [0.83, 1.36]	0.61
	Multivariable: pre-op AHI and Maxillary Advancement adjusted	1.03 [0.82, 1.30]	0.80
	Multivariable: pre-op AHI and Mandibular Advancement adjusted	1.03 [0.81, 1.30]	0.82

## Electronic Supplementary Material

Below is the link to the electronic supplementary material.

Supplementary Material 1

## Data Availability

Data used in this study is available upon request and appropriate steps through institutional IRB.
