# Supplementary material for: Positive airway pressure settings do not predict outcomes of maxillomandibular advancement surgery in the treatment of obstructive sleep apnea
Source: Oral Maxillofac Surg. 2025 Jun 21;29(1):129. doi: 10.1007/s10006-025-01421-8 (PMC12181112; doi:10.1007/s10006-025-01421-8)
Supplement: Supplementary file 1 — Supplementary Material 1 [file 10006_2025_1421_MOESM1_ESM.docx]

**Supplemental Table 1. CPAP values for patients with surgical success and resolution of OSA**

|  | Had outcome | Did not have outcome |
| --- | --- | --- |
| Outcome: Apnea-hypopnea index (AHI) (from CPAP), of < 20 and 50% reduction in AHI |  |  |
| CPAP value, mean (SD) | 10.2 (3.1) | 11.0 (4.5) |
| % with high CPAP value | 79 | 71 |
| Outcome: Apnea-hypopnea index (AHI) (from CPAP), of < 5 |  |  |
| CPAP value, mean (SD) | 10.6 (3.6) | 10.3 (3.4) |
| % with high CPAP value | 78 | 77 |
